# Comparative Investigation of Vortex and Direct Plasma Discharge for Treating Titanium Surface

**DOI:** 10.3390/biomimetics10010007

**Published:** 2024-12-26

**Authors:** Hyun-Jeong Jeon, Subin Seo, Ara Jung, Kyeong-mok Kang, Jeonghoon Lee, Bomi Gweon, Youbong Lim

**Affiliations:** 1 Plasmapp R&D Center, 9, Giheungdanji-ro 24beon-gil, Giheung-gu, Yongin-si 17086, Republic of Korea; hjjeon@plasmapp.com (H.-J.J.); sbseo@plasmapp.com (S.S.); kmkang@plasmapp.com (K.-m.K.); jhlee@plasmapp.com (J.L.); 2 Department of Mechanical Engineering, Sejong University, 209, Neungdong-ro, Gwangjin-gu, Seoul 05006, Republic of Korea; eeyorelove@sejong.ac.kr

**Keywords:** vacuum plasma treatment, dental material, hydrophilicity, plasma cleaning, osteoblast

## Abstract

Numerous studies have investigated the surface treatment of implants using various types of plasma, including atmospheric pressure plasma and vacuum plasma, to remove impurities and increase surface energy, thereby enhancing osseointegration. Most previous studies have focused on generating plasma directly on the implant surface by using the implant as an electrode for plasma discharge. However, plasmas generated under atmospheric and moderate vacuum conditions often have a limited plasma volume, meaning the shape of the electrodes significantly influences the local electric field characteristics, which in turn affects plasma behavior. Consequently, to ensure consistent performance across implants of different sizes and shapes, it is essential to develop a plasma source with discharge characteristics that are unaffected by the treatment target, ensuring uniform exposure. To address this challenge, we developed a novel plasma source, termed “vortex plasma”, which generates uniform plasma using a magnetic field within a controlled space. We then compared the surface treatment efficiency of the vortex plasma to that of conventional direct plasma discharge by evaluating hydrophilicity, surface chemistry, and surface morphology. In addition, to assess the biological outcomes, we examined osteoblast cell activity on both the vortex and direct plasma-treated surfaces. Our results demonstrate that vortex plasma improved hydrophilicity, reduced carbon content, and enhanced osteoblast adhesion and activity to a level comparable to direct plasma, all while maintaining the physical surface structure and morphology.

## 1. Introduction

Various dental materials, including dental implants, abutments, titanium meshes, bone grafts, resin cements, and crowns, are widely used in the treatment of oral diseases, prosthetics, restorations, orthodontics, and aesthetic procedures. Among these, dental implants are essential medical devices used to replace missing teeth and restore functionality. Titanium or titanium alloys are the most commonly used materials for dental implants due to their excellent biocompatibility and strong corrosion resistance, which enable them to withstand the challenging conditions of the oral environment [1]. The most critical factor for the success of implants is their ability to achieve stable fixation through effective osseointegration. To improve the stability of the implanted fixture, the surface characteristics of the implant, particularly those in direct contact with bone tissue, are crucial in enhancing implant stability [2]. Consequently, various surface modification techniques have been developed to improve implant performance. These include methods to create microscale surface roughness or coarser topographies, such as sandblasting with large grit acid etching, laser ablation, and electrochemical anodic oxidation. Additionally, surface coating with biocompatible substances or agents that promote osseointegration, such as proteins and growth factors, has also been explored [3,4,5].

However, over time, the surface of a dental implant may degrade and lose its original biocompatibility from the moment it is manufactured until it is implanted in a patient. This surface aging can occur due to changes in surface chemistry and the accumulation of carbon-based impurities on the implant surface [6]. This accumulation can obscure the initially modified, biologically favorable characteristics, reducing the surface’s hydrophilicity and biological activity, thereby diminishing the intended benefits of surface treatments [6,7].

Previous studies have shown that plasma surface treatment can provide a solution to mitigate the decline in bioactivity caused by carbon accumulation [8,9]. Plasma treatment enhances biocompatibility through several mechanisms, with the primary effect being an improvement in surface hydrophilicity. This is achieved by promoting the formation of oxygen-containing functional groups, such as hydroxyl (OH) groups, on the implant surface, enhancing hydrophilicity [10]. Increased hydrophilicity promotes protein adsorption and cell adhesion after implantation, thereby improving osseointegration [10,11].

Another key mechanism is the reduction of carbon-based impurities (hydrocarbons) on the implant surface. Reactive species and ions in the plasma can remove surface hydrocarbons by reacting with them and converting them to CO_2_ and H_2_O [12,13]. The removal of hydrocarbons from the surface can further enhance the biocompatibility of the implant by exposing the underlying bioactive surface, which in turn can enhance osteoblast activity and bone conductivity [9,14].

Plasma treatment methods for implant surfaces include atmospheric pressure plasma treatment, which utilizes specific gases such as argon, oxygen, and nitrogen in a chamber or jet format, and vacuum plasma treatment [8,9,15,16,17,18]. Vacuum plasma treatment, due to its low discharge voltage, can generate plasma using only air rather than inert gases, eliminating the need for additional gas supply. Furthermore, it produces higher electron energy compared to atmospheric pressure plasma due to the longer acceleration distance of electrons [9]. As a result, vacuum plasma can be more effective at removing impurities from the implant surface. Preclinical and clinical studies have demonstrated that implants treated with vacuum plasma exhibit improved early implant stability, increased bone-to-implant contact, and reduced marginal bone loss, all of which contribute to enhanced osseointegration [14,19].

Similarly, plasma treatment can enhance the surface activity of not only implants but also other dental materials. In particular, custom abutments used after implant placement may become contaminated by titanium wear particles from manufacturing processes and hydrocarbons from storage and air pollutants, which can interfere with cellular activity, delay healing, and increase the risk of infection [20,21]. To address this issue, a study conducted by Canullo et al. (2024) found that treating a titanium disk, made of the same material as the abutment, with vacuum plasma significantly increased epithelial cell attachment and promoted cellular activity on the titanium surfaces [20].

Although the various effects of vacuum plasma have been well documented, conventional vacuum plasma treatments are typically designed to use metallic dental materials as either ground or powered electrodes [8,9,14]. For instance, when the metallic dental material acts as a ground electrode by contacting the electrically grounded components of the plasma treatment device, plasma is discharged by the electric field generated between this ground electrode and a separately powered electrode within the device. Plasma discharge is governed by the electric field, which is, in turn, influenced by the shape and configuration of the electrodes. The shape of the electrode determines the distribution, density, and strength of the electric field, as the electric field lines are always perpendicular to the surface of the conductor. For example, sharp or pointed electrode geometries create regions of higher electric field intensity due to the concentration of field lines, facilitating easier plasma ignition and localized discharge. In contrast, flat or rounded electrodes produce a more uniform but weaker electric field, which may lead to a more homogeneous discharge across the surface. These variations in electric field behavior directly affect plasma characteristics, such as its density, energy distribution, and uniformity [22,23]. Consequently, the structure and shape of the dental material, acting as electrodes, can significantly influence the performance of plasma surface treatments.

Furthermore, uniform plasma treatment can only be achieved when the dental material is perfectly grounded to the device, which limits treatment to metallic materials with uniform shapes. Therefore, processing dental materials with varying shapes and compositions, such as custom abutments, titanium mesh, and non-metallic bone grafts, is challenging.

To address these limitations, in this study, we developed a container enabling indirect plasma treatment of dental materials without requiring direct grounding to the treatment target. Titanium, widely used in dental materials such as implants and abutments, was selected as a test sample. The hydrophilicity, surface chemistry, and surface morphology of the titanium coupons were assessed and compared after direct or indirect plasma treatment. Additionally, osteoblast activity of the plasma-treated surfaces was evaluated to determine any improvements in bioactivity.

## 2. Materials and Methods

### 2.1. Test Sample and Plasma Treatment

**Figure 1:**
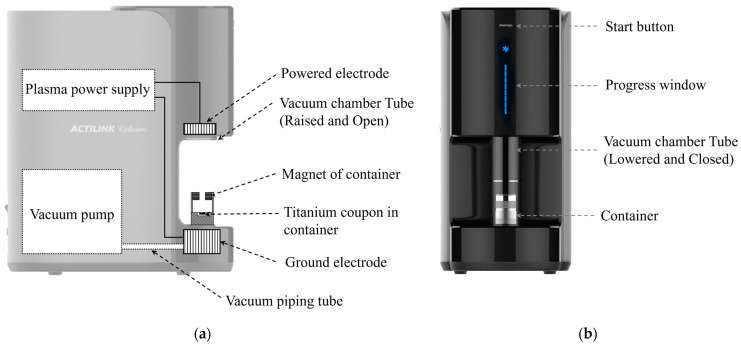
(**a**) Piping and instrument diagram of the plasma activator (side view) and (**b**) device image and key functional elements in the front view.

Titanium coupons with a diameter of 12 mm and a thickness of 4 mm were prepared from commercially pure titanium (grade 4). A vacuum plasma treatment device (ACTILINK reborn, Plasmapp Co., Ltd., Daejeon, Republic of Korea) was used to treat the titanium coupons, which were placed inside a specially designed container, as shown in Figure 1.

Once the device was started by pressing the start button (Figure 1b), a glass tube descended, covering the container and contacting the silicone base at the bottom, forming an airtight seal. Then, the vacuum pumps pumped out the air through the port placed right next to the ground electrode. A diaphragm pump was used for the vacuum plasma treatment device to obtain a base pressure of about 10 torr. A sinusoidal electric power with a frequency of 100 kHz and peak-to-peak voltage of approximately 3 kV was applied to the powered electrode by the plasma power supply, generating a strong electric field to discharge plasma within the vacuum chamber. After the plasma treatment, plasma power was terminated, and the vacuum chamber was purified by continued vacuuming. Then, HEPA-filtered air was introduced to return the vacuum chamber to atmospheric pressure, allowing the vacuum chamber tube to rise.

**Figure 2:**
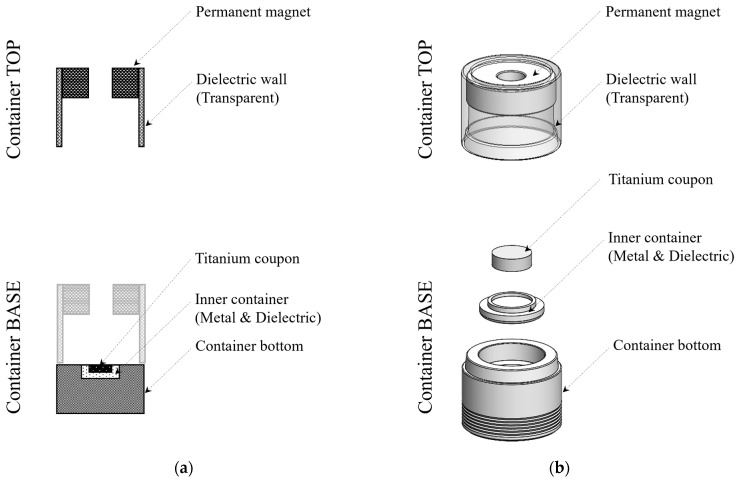
Configuration of the container with (**a**) conceptual and (**b**) actual drawing.

As depicted in Figure 2, the container consists of a base and a top part, with a permanent magnet located in the top part. The magnet was electrically insulated from the container base, which was electrically grounded by placing it on the ground electrode of the plasma treatment device, as shown in Figure 1.

The titanium coupon was placed inside the container using an inner container made of either metal (stainless steel) or dielectric materials (polycarbonate). When the metal inner container was used, the titanium coupon was electrically grounded; when the dielectric inner container was used, the coupon remained electrically floating. When the titanium coupon was grounded, “direct plasma” was discharged directly onto the coupon surface. In this study, the plasma discharge time for the direct plasma treatment was set to 15 s, and it was denoted by D15. When the titanium coupon was floating, plasma was discharged in the form of “vortex plasma”, which indirectly exposed the titanium coupon to plasma. The discharge times for vortex plasma were varied, with durations of 15, 30, and 60 s, denoted as V15, V30, and V60, respectively.

### 2.2. Contact Angle Test

To evaluate the improvement in hydrophilicity resulting from plasma treatment, distilled water was dropped on the surface of the Control (not treated), D15, V15, V30, and V60 samples, and the contact angle was measured using a portable contact angle measurement tool (Aqua-K1, PSM, Seongnam, Republic of Korea) and its associated software, PSM CAM Ver1.0. For the plasma-treated group, the contact angle was measured immediately after plasma treatment. Each test was performed in triplicate for each condition.

### 2.3. X-Ray Photoelectron Spectroscopy Analysis

The surface of the titanium coupon was analyzed by X-ray photoelectron spectroscopy (XPS, PHI 5000 VersaProbe III, ULVACPHI Inc., Chigasaki, Japan) to measure the chemical composition of the titanium surface. The X-ray source was monochromated Al Kα (1486.6 eV), and the beam spot size and power were set to 100 µm and 25.33 W, respectively. The scan pass energy was set to 224 eV (eV step:0.8). The XPS analysis was performed on the titanium coupons of the Control, D15, V15, V30, and V60, with measurements taken at three locations (center and both sides) on each coupon.

### 2.4. Surface Observation by Scanning Electron Microscopy

The surface morphology was observed using scanning electron microscopy (SEM, 4000Pro, CIQTEK, Hefei, China). The SEM observation conditions were as follows: Schottky Field Emission tip was used as the beam source, the acceleration voltage was set to 15 kV, and the image mode was set to high-vacuum SE mode.

The surface of the titanium coupon before plasma treatment was analyzed with SEM, and after which the coupon was treated using the plasma device (ACTILINK reborn), with the direct plasma treatment for 15 s or the vortex plasma treatment for 30 s. After the plasma treatment, the surface of the titanium coupon was reanalyzed with SEM at the same location as before the plasma treatment. To determine surface variation by sample location, the SEM observation sites were selected as the center and both sides of each sample.

### 2.5. Measuring Surface Roughness with Atomic Force Microscopy

The roughness of the titanium surface without and with plasma treatment was measured using atomic force microscopy (AFM, XE-100, Park Systems, Suwon, Republic of Korea). The surface roughness was measured for the Control sample (without plasma treatment), the D15 sample (treated with direct plasma for 15 s), and the V30 sample (treated with vortex plasma for 30 s). For each sample, roughness was measured for a 20 μm × 20 μm area at the center and both sides, i.e., three positions per sample, and the average value was used to determine the roughness of each sample.

### 2.6. Alkaline Phosphatase (ALP) Activity Test

ALP activity test was performed using Saos-2 cells (#80023, Korean cell line bank, Seoul, Republic of Korea) to determine the effect of the vacuum plasma surface treatment on cell differentiation. Saos-2 cells were cultured in Minimum Essential Medium (#LM007-01, Welgen, Gyeongsan, Republic of Korea) with 10% fetal bovine serum (FBS, #S001-01, Sigma, Saint Louis, MI, USA) and 1% Antibiotic-Antimycotic (Anti-Anti #CA002-010, GenDEPOT, Katy, TX, USA). The titanium coupon of the Control, D15, and V30 were placed on a 24-well plate, and Saos-2 was seeded at 6 × 10^4^ cells/well and cultured in a CO_2_ incubator at 37 °C for 7 days. After the culture period, the ALP activity of cells attached to the titanium coupon was measured according to the ALP test kit (#ab83369, Abcam, Cambridge, UK). The absorbance for the ALP activity test was measured at 405 nm using a microplate reader (AMR-100, Allsheng, Hangzhou, China). The experiments were repeated five times for each condition.

### 2.7. Fluorescence Cell Imaging

Titanium coupons for the Control, D15, and V30 groups were placed in a 24-well plate, and Saos-2 cells were seeded at a density of 3 × 10^5^ cells/well. The cells were cultured in a CO_2_ incubator at 37 °C for 1 day. Following incubation, the cells were fixed in 4% paraformaldehyde solution for 10 min and then permeabilized with 0.2% Triton X-100 in phosphate-buffered saline (PBS) for 15 min at room temperature. At each step, the samples were washed three times with PBS.

To label actin stress fibers, the cells on the titanium coupons were stained using Rhodamine phalloidin (#R415, Invitrogen, New York, NY, USA; 1:50 dilution in PBS). For nuclei staining, 5 µg/mL Hoechst 33342 (#H3570; Invitrogen) diluted in PBS was used. A drop of mounting solution (PROLONG GLASS, #P36980, Invitrogen) was applied to glass-bottom dishes (#101350; SPL Life Sciences, Pocheon, Republic of Korea), and the titanium coupons were placed on the dishes with the cell-attached surface facing downward. Fluorescence images were acquired using an inverted wide-field fluorescence microscope (DMi8; Leica Microsystems, Wetzlar, Germany).

## 3. Results

### 3.1. Plasma Treatment

**Figure 3:**
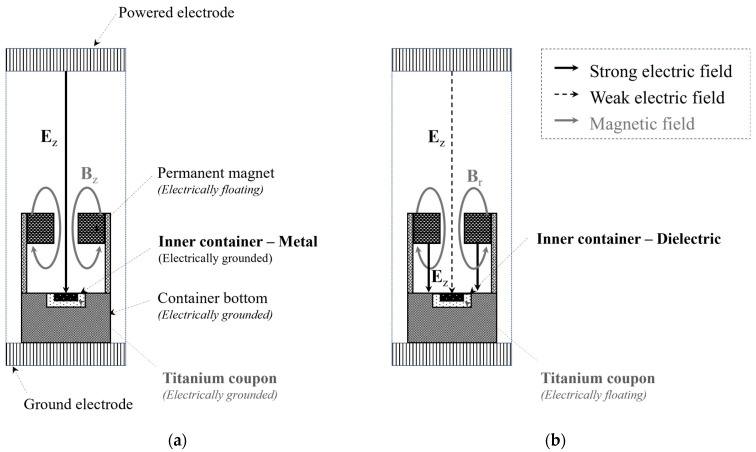
Configurations of electric and magnetic field for (**a**) direct and (**b**) vortex plasma discharge.

When the titanium coupon was electrically grounded using the metal inner container, the axial electric field was generated directly on the surface of the titanium coupon, as shown in Figure 3a. Plasma was then discharged onto the titanium surface along the direction of this strong electric field.

When the titanium coupon was electrically floating within a dielectric inner container, a strong electric field was generated between the grounded container bottom and the floating magnet, as depicted in Figure 3b. The electric potential of the floating magnet was determined by the discharged plasma in the vacuum chamber, and the electric field was defined by the potential difference between the magnet’s potential and the grounded potential of the container bottom. Notably, a weak electric field was generated on the titanium coupon, as the electric potential of the coupon was influenced by the plasma discharged by the strong electric field around the inner container.

As shown in Figure 3, the container has a permanent magnet, which generates a magnetic field inside the container. Direct plasma was discharged by the axial electric field where the direction of the magnetic field was parallel to the electric field, as depicted in Figure 3a. The vortex plasma was generated by strong electric and magnetic fields perpendicular to each other, in which the electric and magnetic fields are mainly formed by axial and radial directions, respectively. Indeed, the interaction of these **E × B** fields is fundamental to generating the uniform plasma within the container. When the rotation speed of this vortex plasma is sufficiently high, it results in a spatially uniform plasma distribution within the container and around the titanium coupon.

**Figure 4:**
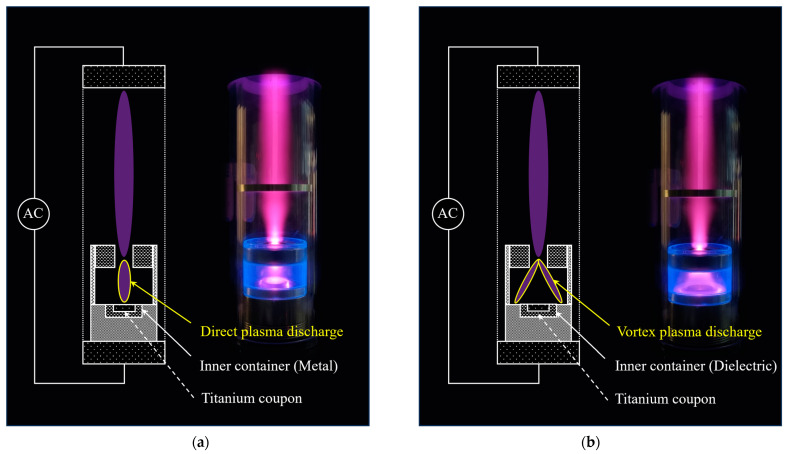
Discharge configurations and image for (**a**) direct and (**b**) vortex plasma.

The vacuum chamber tube and side wall of the container were designed to be transparent, allowing the plasma discharged inside the container to be directly observed from the outside. The resulting plasma discharge images are shown in Figure 4.

The direct plasma was discharged between the powered electrode and the titanium coupon, as illustrated in Figure 4a. A strong magnetic field was generated at the center of the magnet, leading to a higher plasma density at the hole of the magnet, as shown in both Figure 4a,b. Notably, the shape of the direct plasma discharge did not appear to be significantly influenced by the magnetic field, as the magnetic field was aligned parallel to the electric field, as shown in Figure 3a.

Figure 4b shows the discharge configuration and discharge image of the vortex plasma, which was discharged between the magnet and container bottom. Inside the container, the plasma was rotated by the magnetic field, with a rotation speed sufficiently high to create spatially uniform plasma around the titanium coupon. A video illustrating the discharge behavior of the vortex plasma is provided as a video in the Supplementary Materials (Video S1). The titanium coupons of the V15, V30, and V60 were treated with vortex plasma for discharge times of 15, 30, and 60 s, respectively.

### 3.2. Hydrophilicity of the Titanium Sample

**Figure 5:**
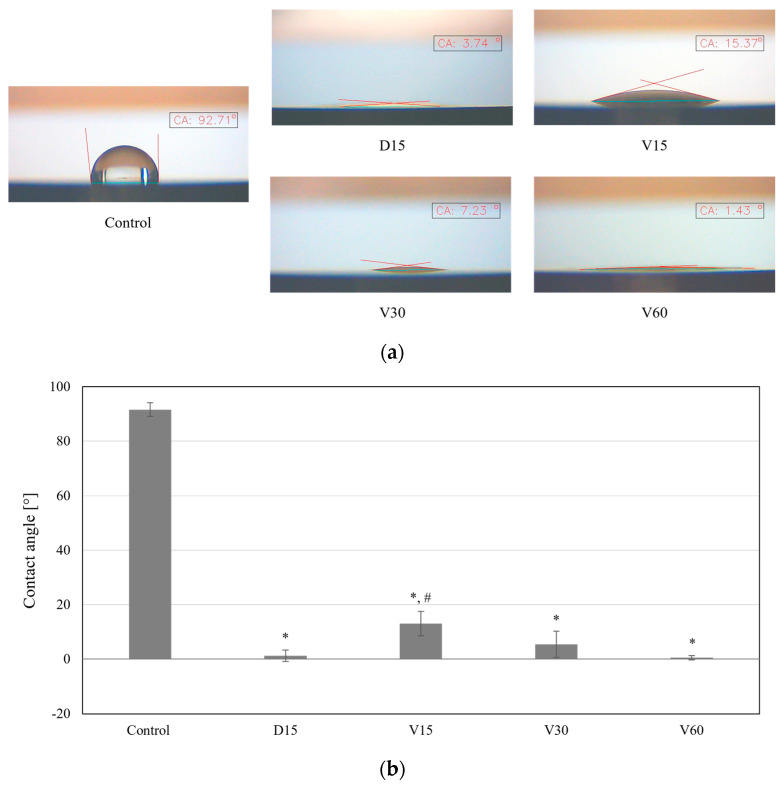
(**a**) Representative images of the contact angle measured for each sample and (**b**) measurement results of the contact angle. *, *p* < 0.001; #, *p* < 0.05 (Unpaired student’s *t*-test. * mark was compared to the Control, and # mark was compared to D15.).

The improved hydrophilicity of the titanium surface after plasma treatment was confirmed by measuring the contact angle, as shown in Figure 5. The non-treated (Control) titanium surface had a contact angle of 91.6 ± 2.5°. As shown in Figure 5a, the hydrophilicity of the titanium surface was significantly enhanced following vacuum plasma surface treatment. The contact angle of the D15, treated with the direct plasma discharge, was measured to be 1.2 ± 2.2°, indicating superhydrophilicity. The contact angles for the titanium coupons treated with vortex plasma for 15, 30, and 60 s (V15, V30, and V60) were measured as 13 ± 4.4°, 5.4 ± 4.8°, and 0.5 ± 0.8°, respectively. Notably, the titanium surface displayed progressively improved hydrophilicity with longer vortex plasma discharge times.

### 3.3. Elemental Content Analysis on the Titanium Surface

**Figure 6:**
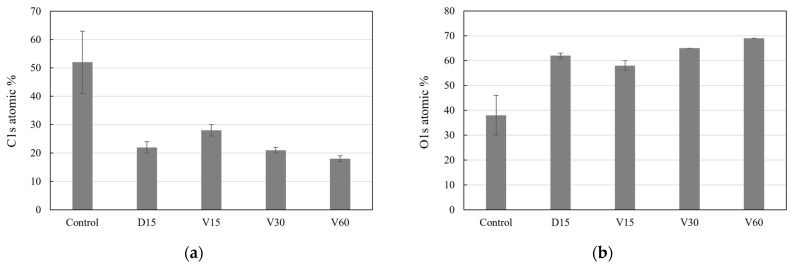
Chemical composition of (**a**) carbon and (**b**) oxygen on the titanium surface.

The XPS analysis was performed on the titanium coupons of the Control, D15, V15, V30, and V60 to determine the effect of the plasma surface treatment, and the chemical compositions of carbon, oxygen, and titanium were obtained. XPS spectra at each analysis location of the samples are provided as Supplementary Materials (Figure S1). The carbon and oxygen levels were significantly affected by the vacuum plasma treatment, as shown in Figure 6.

The carbon level of the Control was measured to be 52 ± 11 at%, and it was significantly reduced by the direct and vortex plasma surface treatment, as shown in Figure 6a. The carbon level was significantly reduced by direct plasma to be 22 ± 2 at%, which was about 58% less than before the plasma treatment. The effect of vortex plasma treatment on carbon reduction was investigated with different discharge times of 15, 30, and 60 s. The carbon levels of the titanium coupons of the V15, V30, and V60 were measured to be 28 ± 2 at%, 21 ± 1 at%, and 18 ± 1 at%, respectively. As the discharge time of the vortex plasma treatment was increased, the carbon level was reduced by 46% (V15), 60% (V30), and 65% (V60) compared to the Control. It was noted that a similar carbon reduction performance was obtained for the vortex plasma with a discharge time of 30 s (V30) as the direct plasma with a discharge time of 15 s (D15).

Figure 6b presents the measurement results of the oxygen level of the titanium coupons. The oxygen level was found to be significantly increased by the vacuum plasma treatment. The oxygen level of the Control was measured to be 38 ± 8 at%, while it was found to be 62 ± 1 at% for the D15. The V15, V30, and V60 have an oxygen level of 58 ± 2 at%, 65 at%, and 69 at%, respectively. It was observed that the oxygen composition on the titanium surface increased with longer discharge times. The titanium level was slightly increased by the vacuum plasma treatment, with levels of 10 ± 2 at%, 16 ± 2 at%, 14 ± 1 at%, 14 ± 1 at%, and 13 ± 1 at% for the Control, D15, V15, V30, and V 60, respectively. It was noteworthy that the sum of the chemical composition of carbon, oxygen, and titanium was approximately 100%.

**Figure 7:**
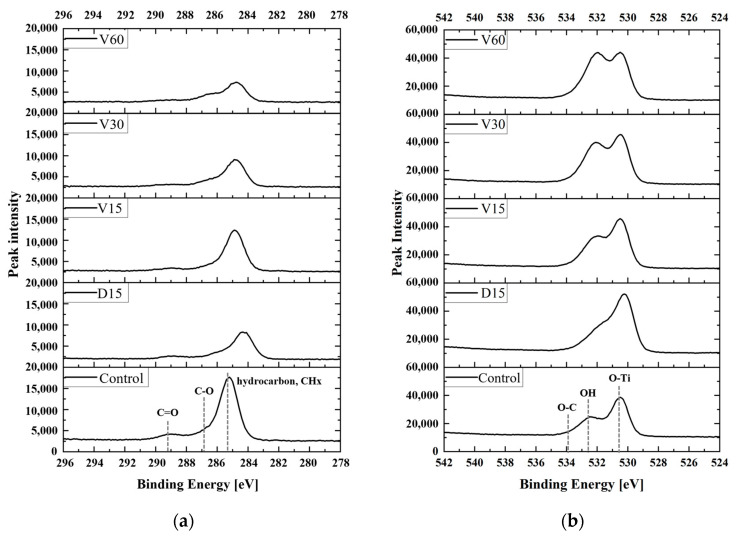
(**a**) C1s and (**b**) O1s narrow peaks measured on the surface of each sample.

To investigate the trends in carbon and oxygen changes under different plasma treatment conditions, the narrow peaks of C1s and O1s at the center of each sample were analyzed and are presented in Figure 7. Plasma treatment resulted in a significant reduction of the hydrocarbon peak in the C1s spectrum, while the O-Ti bond intensity in the O1s spectrum generally increased. Additionally, for vortex plasma treatment, it was observed that with longer treatment times, the C1s peak progressively decreased, while the intensity of the OH peak tended to increase.

**Figure 8:**
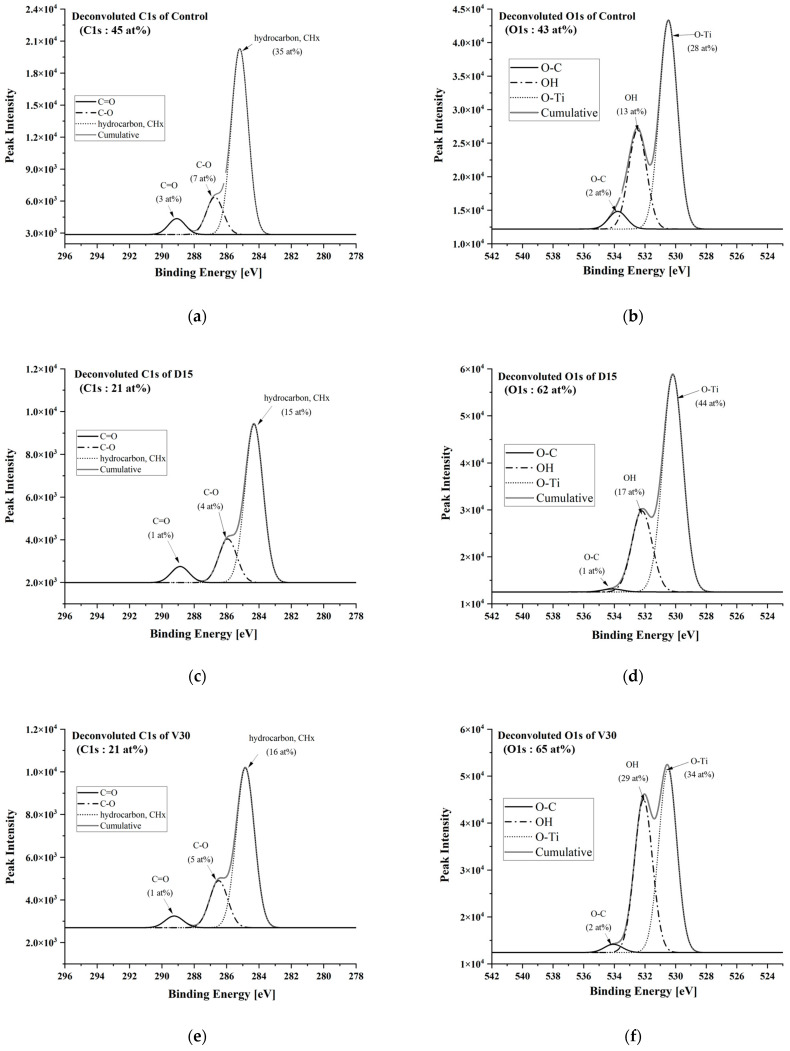
Deconvoluted results of the (**a**,**c**,**e**) C1s and (**b**,**d**,**f**) O1s peak. (**a**,**b**) Control, (**c**,**d**) D15, and (**e**,**f**) V30 sample.

To further investigate the surface chemical composition of D15 and V30, which exhibited similar hydrophilicity and carbon removal performance, the C1s and O1s peaks at the center of each sample were deconvoluted. Results are presented in Figure 8 and Table 1. In the Control, the Hydrocarbon (CHx) content was 35 at%, while in D15 and V30, it was 15 at% and 16 at%, respectively, indicating similar levels of hydrocarbon reduction. This suggests that the decrease in the C1s peak due to plasma treatment was primarily associated with the reduction of hydrocarbon (CHx) species rather than the C=O and C-O species.

Analysis of the O1s peak revealed that the O-Ti bond intensity was 28 at% in the Control, and it significantly increased to 44 at% and 34 at% in D15 and V30, respectively. Notably, this increase was more pronounced in D15. Furthermore, the OH peak intensity increased from 13 at% in the Control to 17 at% and 29 at% in D15 and V30, respectively. The increase was particularly more prominent in V30.

### 3.4. Surface Morphology and Roughness

**Figure 9:**
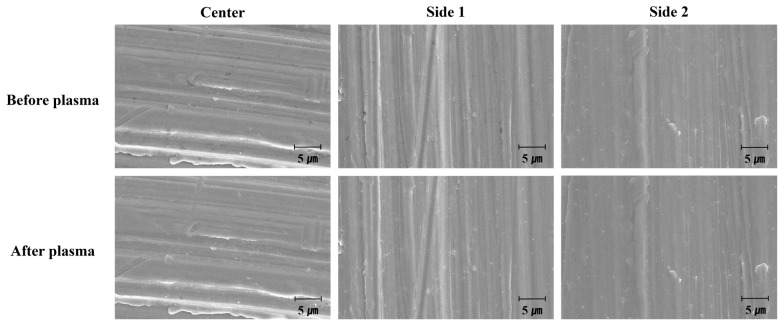
SEM observation of the surface by position on the titanium coupon before and after treatment with direct plasma for 15 s (10,000×).

**Figure 10:**
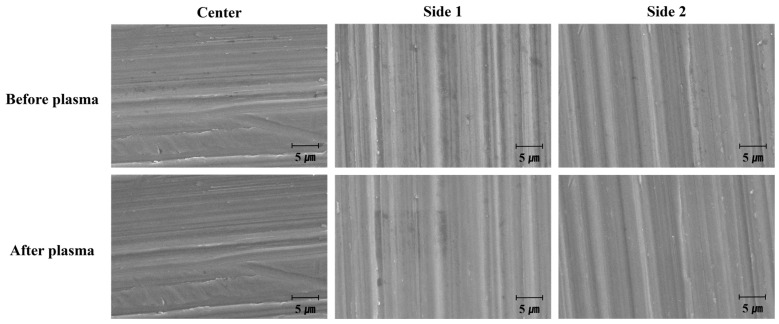
SEM observation of the surface by position on the titanium coupon before and after treatment with vortex plasma for 30 s (10,000×).

To evaluate the effect of plasma treatment on the morphology of the titanium coupon surface, SEM observations were conducted at the same surface location both before and after plasma treatment. SEM images of the center and sides of the titanium coupon revealed that after 15 s of direct plasma treatment (Figure 9), the black areas, likely representing organic compounds (presumed carbon-based impurities), were reduced. However, no significant changes in surface morphology were observed. Similar results were obtained for the titanium coupons treated with vortex plasma for 30 s (Figure 10).

Observations of the surface at magnifications of 5000× and 20,000× before and after treatment with both types of plasma showed no significant changes in the morphology of the titanium surface (Figure S2 in Supplementary Materials). Overall, these findings indicate that neither direct nor vortex plasma treatment caused detectable changes to the morphology of the titanium coupon surface.

The change in surface roughness of the titanium coupons due to plasma treatment was measured using AFM, and the results are presented in Table 2. The roughness (Ra) values of the titanium coupons treated with direct plasma for 15 s (D15) or vortex plasma for 30 s (V30) were not significantly different from those of the Control sample. This indicates that plasma treatment did not alter the intrinsic roughness of the titanium coupons.

### 3.5. Cellular Activity and Distribution on Titanium Surface

**Figure 11:**
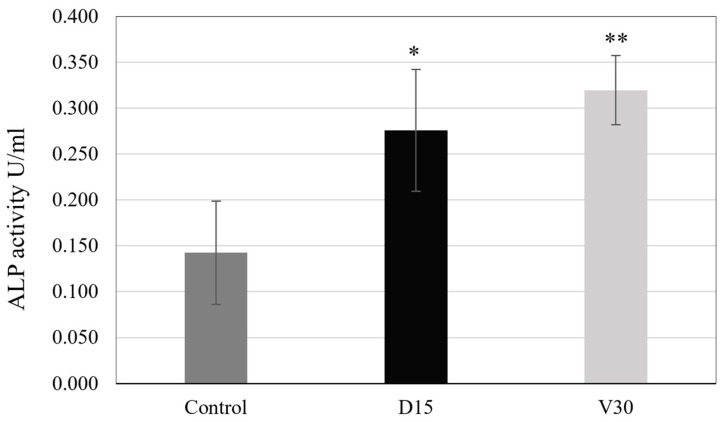
ALP Activity on the titanium coupons of the Control, D15, and V30. * *p* < 0.01, ** *p* < 0.001 (Unpaired student’s *t*-test. Data were compared to the Control).

As shown above, both D15 and V30 plasma-treated titanium coupons exhibited similar hydrophilicity and carbon reduction. To investigate how these surface changes affect osteoblast activity, the ALP activity of Saos-2 cells attached to the Control, D15, and V30 titanium coupons was measured. The ALP activity values for Saos-2 cells on Control, D15, and V30 surfaces are presented in Figure 11. The measured ALP activity values were 0.142 ± 0.038 U/mL for the Control, 0.276 ± 0.071 U/mL for D15, and 0.320 ± 0.041 U/mL for V30. These values represent a 94% for D15 and a 125% increase for V30, both of which are statistically significant, as shown in the figure.

**Figure 12:**
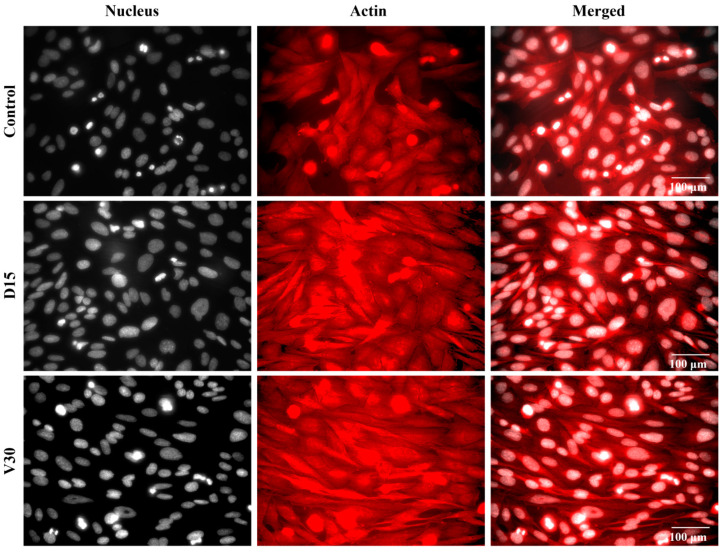
Representative fluorescence microscopy images of Saos-2 cells adhered to the coupon surfaces for the Control, D15, and V30 groups. The nuclei are shown in grayscale, while actin filaments are shown in red. Scale bar = 100 μm.

Cell adhesion on the titanium coupon surfaces was further examined. To visualize cellular structures, cells were labeled with phalloidin to label actin filaments, which is shown in red in Figure 12. Notably, cells on both plasma-treated surfaces (D15 and V30) exhibited a more elongated morphology compared to those on the Control surface. Furthermore, the cellular spreading area was larger on the plasma-treated surfaces (D15 and V30) than on the Control surface.

## 4. Discussion

In this study, an effective vacuum “Vortex plasma” treatment method was developed to enhance the biocompatibility of various dental materials. Titanium is widely used in dental applications due to its high strength-to-weight ratio, mechanical durability, chemical stability, and resistance to corrosion. Furthermore, its elastic modulus, which closely resembles that of bone compared to many other metals, is beneficial for reducing the risk of stress shielding effects. These properties make titanium a preferred material for various dental applications, including the fabrication of endodontic files, orthodontic wires and brackets, and dental implants [24]. In this study, titanium coupons were used as a substitute for various metallic dental materials to evaluate and compare surface modification resulting from both vortex and direct plasma treatment. Additionally, osteoblast (Saos-2) activity was assessed.

Our results demonstrate that both the direct and vortex plasma treatments significantly enhance the hydrophilicity of titanium surfaces. Direct plasma achieved a high level of hydrophilicity with a relatively short plasma treatment time of 15 s. Vortex plasma also significantly increased hydrophilicity compared to the Control, although an additional 15 s was required to reach a hydrophilicity level comparable to that of direct plasma treated for 15 s. These findings confirm that both methods are effective for improving surface hydrophilicity, with vortex plasma requiring a slightly longer treatment time to reach similar results.

XPS analysis of the atomic composition of plasma-treated titanium surfaces revealed a significant reduction in carbon content compared to the Control, along with a corresponding increase in titanium. In particular, after plasma treatment, the removal of hydrocarbons (CHx) was found to be most significant. Reactive oxygen species generated during plasma treatment are known to react with hydrocarbons, converting them into carbon dioxide (CO_2_) and water (H_2_O) [13]. The plasma treatment in this study was conducted in an environment where air, predominantly composed of nitrogen and oxygen, introduced abundant reactive oxygen species into the plasma. In addition, it is known that plasma generated at approximately 10 torr pressure exhibits a high electron density. These high-energy electrons are also known to have sufficient energy to break hydrocarbon bonds [9]. Given that our plasma was operated at approximately 10 Torr, it is likely that these high-energy electrons also contributed to the breakdown of hydrocarbons on the target surfaces. Collectively, these results indicate that plasma treatment effectively removes surface hydrocarbons, revealing the titanium surface. The carbon removal efficiency of both D15 and V30 was found to be comparable, aligning with the results of the hydrophilicity test.

In addition, all plasma treated samples exhibited an increase in the atomic ratio of oxygen compared to the Control. This increase was attributed to the formation of hydroxyl groups (OH) and the oxidation of the titanium surface, which led to an increase in O-Ti bonds as a result of plasma treatment. Water molecules present on the implant surface can adsorb on a TiO_2_ surface both molecularly and dissociatively, with dissociative adsorption leading to the dissociation of an H_2_O molecule into OH and H atoms. [25]. Accordingly, oxygen ions from oxygen and water molecules likely served as the sources for the oxidation of the titanium and the generation of OH groups on the titanium surface. Lin et al. (2018) demonstrated that the hydrophilicity of titanium increases with its degree of oxidation, and Kim et al. (2009) reported that the formation of hydroxyl groups (OH) on TiO_2_ surfaces increases with plasma treatment time, thereby enhancing hydrophilicity [10,26]. As a result, the increase in oxygen-containing chemical structures resulting from plasma treatment appears to have enhanced the hydrophilicity of the titanium surface.

While the O1s atomic percentages for both direct and vortex plasma treatments were similar, the chemical structures formed post-treatment exhibited notable differences. Direct plasma treatment resulted in a relatively higher formation of O-Ti bond, whereas vortex plasma treatment predominantly produced OH groups. This variation is likely attributable to subtle differences in the inherent properties of the two plasma configurations.

Both the surface modification and carbon removal performance tests demonstrated that vortex plasma required at least 30 s of treatment to achieve a level of performance comparable to 15 s of direct plasma treatment. This difference is likely due to the distinct plasma generation mechanisms. In direct plasma treatment, the plasma is generated directly on the titanium surface, while in vortex plasma, the plasma is generated between the magnet on the container’s top and the bottom edge of the container. The plasma expands into the surrounding space before reaching the titanium surface. As a result, the density and energy of the reactive species in vortex plasma may be lower, requiring a longer treatment time to achieve similar performance.

Increased hydrophilicity and the reduction of carbon on the implant surface through plasma treatment are known to positively impact cell attachment and differentiation by enhancing protein binding to the implant surface [8]. Studies using a beagle model have shown that this improvement in cell attachment and differentiation can increase bone-to-implant contact and bone volume, thereby promoting osseointegration, a key function of implants [27]. In this study, to compare the potential effects of the two plasma treatment modes on osseointegration, the differentiation performance of Saos-2 cells on titanium samples treated with each plasma method was assessed. Based on the hydrophilicity and carbon removal performance results, D15 and V30 conditions were selected for comparison, as they demonstrated similar performance. ALP activity is a marker for osteogenic function that is well established, with higher ALP activity indicating greater osteoblast differentiation and maturation. Our results showed that the ALP activity was significantly higher in the plasma-treated groups compared to the Control, confirming that both plasma treatment methods enhance osteoblast activity. Moreover, no significant difference in ALP activity was observed between the D15 and V30 treatments, which is consistent with their comparable effects on hydrophilicity and carbon removal.

Furthermore, the cellular morphology on the titanium coupons indicated that the plasma-treated surfaces (D15 and V30) promoted better cell spreading compared to the control group. These findings suggest that both plasma treatments enhanced the titanium surface’s affinity for osteoblasts, thereby increasing the activity of osteoblasts adhering to the surface.

When plasma is applied to dental material surfaces to enhance their biocompatibility, preserving the physical integrity and topography of the material is one of the most important factors. This is particularly important for dental materials in direct contact with bones or gum tissues, as they may have specialized coatings or surface treatments, depending on the manufacturer, to promote interaction with osteoblasts or epithelial cells [4]. In our previous study, we confirmed that the vacuum plasma treatment using the dielectric barrier discharge method did not alter the calcium content on calcium-coated implants [8]. Similarly, in this study, the surface analysis of titanium coupons before and after plasma treatment using SEM and AFM revealed that neither direct plasma treatment nor vortex plasma treatment resulted in any significant changes to the morphology or roughness of the titanium surface, indicating that the unique physical properties of titanium were preserved.

To apply direct plasma treatment effectively, the dental material must be perfectly grounded to the plasma treatment container. However, many dental materials, even those made of metal, may not achieve perfect grounding due to their irregular shapes, such as custom abutments and titanium mesh, resulting in uneven plasma treatment across the surface. Additionally, non-conductive materials, such as bone grafts, are challenging to treat with direct plasma. In contrast, vortex plasma treatment offers a convenient and effective alternative for enhancing the biocompatibility of various dental and medical materials, as it ensures uniform plasma treatment across the entire surface, regardless of the material’s shape or conductivity. Further studies would be valuable in assessing the effects of vortex plasma on a broader range of materials.

## 5. Conclusions

In this study, the vortex plasma treatment method was developed to indirectly and uniformly treat the surfaces of dental materials to improve their biocompatibility. The performance of vortex plasma treatment was compared with that of direct plasma treatment. Titanium, widely used in dental implants, was selected to evaluate surface modifications and osteoblast activity following both plasma treatments. Both treatments rapidly improved the hydrophilicity of the titanium surface within 15 s, and significantly reduced surface carbon content without causing any physical damage to the titanium. However, because vortex plasma does not directly interact with the titanium surface but instead exposes it indirectly, more treatment time was required to achieve comparable hydrophilicity and carbon removal performance to direct plasma. Specifically, 15 s of direct plasma treatment was equivalent to 30 s of vortex plasma treatment. The increased hydrophilicity and reduction in surface impurities resulting from both plasma treatments significantly enhanced osteoblast activity on the titanium surface. This improvement in cell attachment and differentiation is expected to positively influence osseointegration during actual implant placement. Notably, vortex plasma is anticipated to be particularly useful for increasing the biocompatibility of metal products with irregular shapes or dental materials that are non-conductive.

## Figures and Tables

**Table 1:** Atomic % of the deconvoluted C1s and O1s Peaks from XPS analysis [at%].

Peak	Control	D15	V30
C1s	45	21	21
C=O	3	1	1
C-O	7	4	5
CHx	35	15	16
O1s	43	62	65
O-C	2	1	2
OH	13	17	29
O-Ti	28	44	34

**Table 2:** Ra results of each sample measured by AFM [μm].

Control	D15	V30
0.98 ± 0.19	0.93 ± 0.23	1.01 ± 0.67

## Data Availability

All the data are presented in this manuscript.

## Supplementary Materials

The following supporting information can be downloaded at https://www.mdpi.com/article/10.3390/biomimetics10010007/s1: Figure S1: XPS spectra; Figure S2: SEM images; Video S1: The discharge appearance of the vortex plasma.
