# Supplementary figures and images for: Comparative Investigation of Vortex and Direct Plasma Discharge for Treating Titanium Surface
Source: Biomimetics (Basel). 2024 Dec 26;10(1):7. doi: 10.3390/biomimetics10010007 (PMC11759839; doi:10.3390/biomimetics10010007)

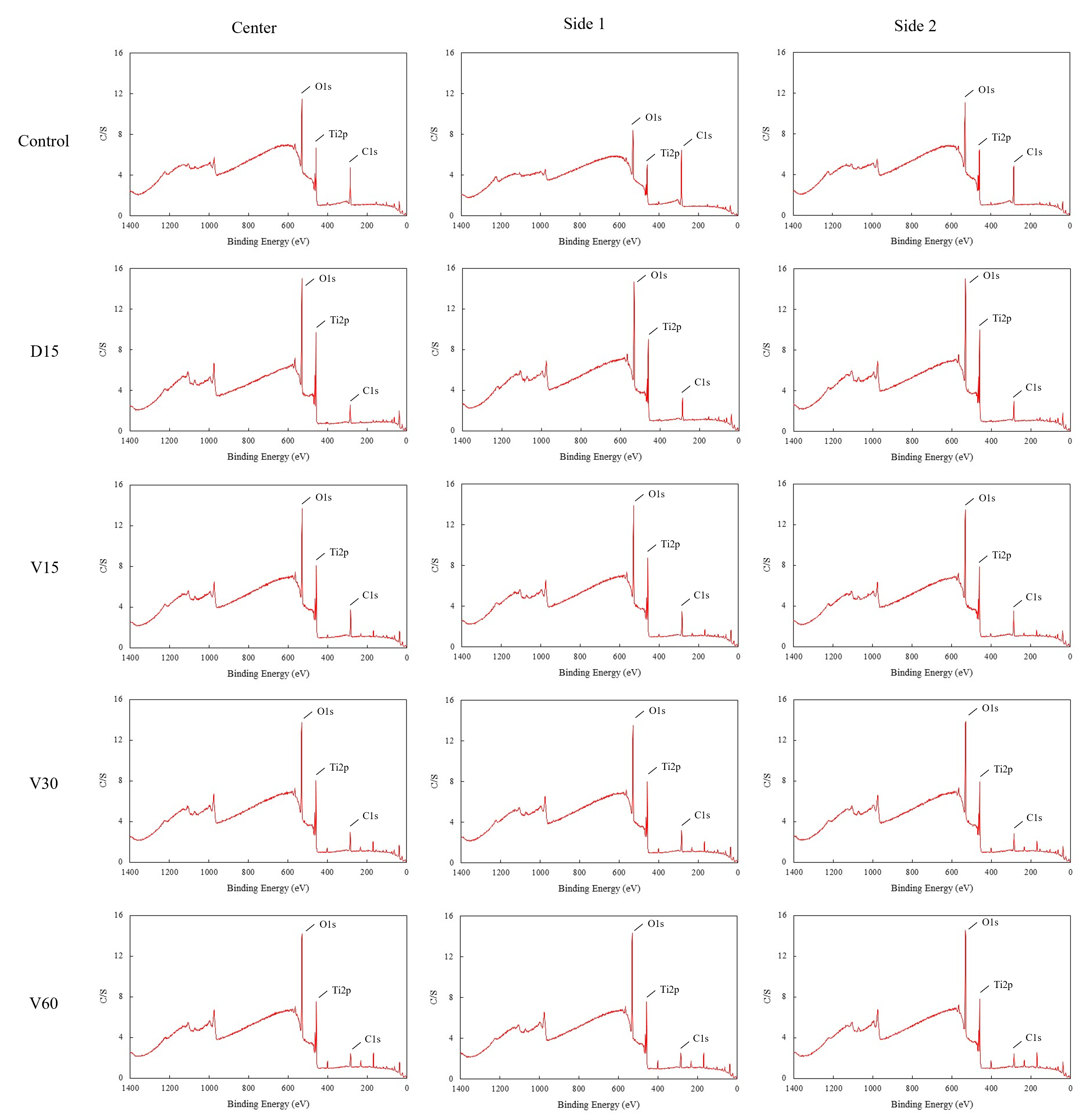

Supplement: Supplementary file 1 [file biomimetics-10-00007-s001.zip › Figure S1. XPS spectra.tif]

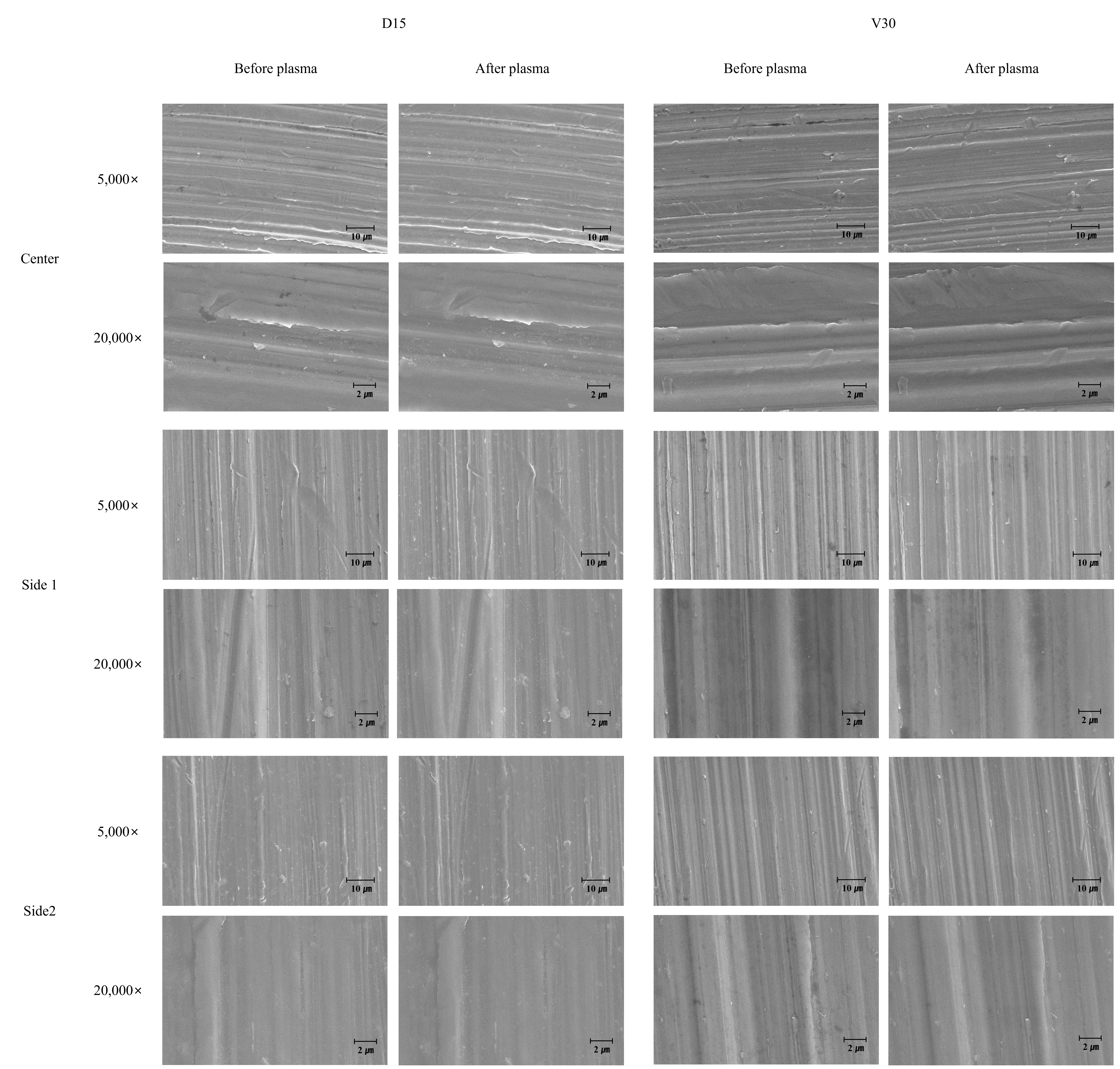

Supplement: Supplementary file 1 [file biomimetics-10-00007-s001.zip › Figure S2. SEM images.tif]
